# Rottlerin exhibits antitumor activity via down-regulation of TAZ in non-small cell lung cancer

**DOI:** 10.18632/oncotarget.13974

**Published:** 2016-12-16

**Authors:** Zhe Zhao, Nana Zheng, Lixia Wang, Yingying Hou, Xiuxia Zhou, Zhiwei Wang

**Affiliations:** ^1^ The Cyrus Tang Hematology Center and Collaborative Innovation Center of Hematology, Jiangsu Institute of Hematology, The First Affiliated Hospital, Soochow University, Suzhou 215123, China

**Keywords:** rottlerin, lung cancer, TAZ, proliferation, apoptosis

## Abstract

Rottlerin, a polyphenolic compound derived from *Mallotus philipinensis*, has been reported to exhibit anti-tumor activities in a variety of human malignancies including NSCLC (non-small cell lung cancer). TAZ (transcriptional co-activator with PDZ-binding motif), one of the key activators in Hippo pathway, has been characterized as an oncoprotein. Therefore, inhibition of TAZ could be useful for the treatment of human cancers. In the current study, we aimed to explore whether rottlerin inhibits the expression of TAZ in NSCLC, leading to its anti-cancer activity. Multiple approaches were applied for determining the mechanism of rottlerin-mediated anti-tumor function, including cell growth assay, Flow cytometry, wound healing assay, invasion assay, Western blotting, and transfection. We found that rottlerin inhibited cell growth, triggered apoptosis, arrested cell cycle, and retarded cell invasion in NSCLC cells. Moreover, our results showed that overexpression of TAZ enhanced cell growth, stimulated apoptosis, and promoted cell migration and invasion. Consistently, inhibition of TAZ exhibited anti-tumor activity in NSCLC cells. Notably, we validated that rottlerin exerted its tumor suppressive function via inactivation of TAZ in NSCLC cells. Taken together, our study indicates that inhibition of TAZ by rottlerin could be a promising strategy for the prevention and therapy of NSCLC.

## INTRODUCTION

In the United States, lung cancer is the leading cause of cancer death in both men and women [1]. The non-small cell lung cancer (NSCLC) accounts for 80–85% of those deaths [2]. Multiple treatments have been applied for NSCLC patients including pneumonectomy, radiation, chemotherapy, or combined modalities [3, 4]. Although these advanced surgical techniques and managements have improved the survival rate, the survival of this disease remains unsatisfactory. Thus, identifying new potential therapeutic agents that inhibit the progression of NSCLC is necessary for the treatment of NSCLC. It has been known that about 25%–48% of current approved therapies for cancer patients by the FDA (Food and Drug Administration) are derived from plants [5, 6]. According to statistics, hundreds of anticancer agents are either pure natural products, natural product derivatives or synthetic compounds with pharmacophores mimicking natural products [7]. Thus, natural products could be considered as a potential source of new anticancer drugs to combat NSCLC.

**Figure 1:**
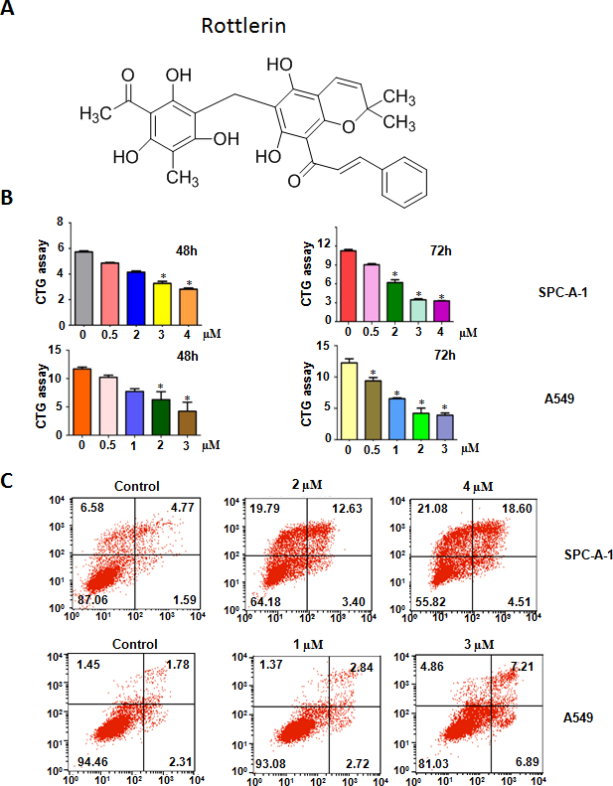
Effect of rottlerin on cell growth, and apoptosis (**A**) The chemical structures of Rottlerin. (**B**) Effect of rottlerin on cell growth in NSCLC was detected by CTG assay. *P < 0.05, compared to the control group (DMSO treatment). (**C**) Cell apoptosis in NSCLC cells treated with rottlerin was determined by Flow cytometry.

Rottlerin, also called mallotoxin, is a natural compound isolated from the kamala tree (Mallotus philippinensis) (Figure 1A) [8]. Increased evidence implicates that rottlerin exhibits pleiorropic anti-tumor activities through promoting apoptosis, autophagy, anti-proliferation, anti-metastasis, and anti-invasion [9]. Several studies have identified rottlerin as an inhibitor of PKC-δ (protein kinase C-delta) in human cancers [10, 11]. Further investigations revealed that rottlerin could inhibit tumor growth via independent of PKC-δ in various types of cancers [12, 13]. For instance, rottlerin enhanced imatinib-triggered apoptosis via its mitochondrial uncoupling effect in BCR/ABL-expressing cells [12]. Lim et al. reported that rottlerin induced apoptosis through upregulation of DR5 (death receptor 5) in CHOP (CCAAT/enhancer-binding protein homologous protein)-dependent manner in human malignant tumor cells [13]. Similarly, rottlerin was reported to down-regulate caspase-2 expression via PKC-δ-independent pathway [14]. The inhibition of P13K (phosphoinosmde-3-kinase)/Akt/mTOR (mammalian target of rapamycin) pathway induced by rottlerin is potential mechanisms for rottlerin-mediated apoptosis [15]. LRP6 (lipoprotein receptor-related protein-6) was inhibited by rottlerin, leading to anti-proliferation and promoting apoptosis in prostate and breast cancer cells [16]. One study demonstrated that rottlerin inhibited lonicera japonica-induced photokilling through cytoskeleton-related signaling cascade in human lung cancer cells [17]. Although some studies have determined the molecular insight onto rottlerin-mediated tumor suppressive function, the underlying mechanisms have still unclear in NSCLC.

The Hippo pathway, an evolutionally conserved signaling cascade, regulates organ size and stem cell property via deregulation of stem cell proliferation and apoptosis [18, 19]. YAP (Yes associated protein) and TAZ (transcriptional co-activator with PDZ-binding motif) are two key targets and terminal effectors, which mediate the biological functions of the Hippo pathway [20]. TAZ has been proved to connect with diverse cellular functions such as differentiation, cell cycle progression, and apoptosis [21]. Over-expression of TAZ can accelerate tumor growth and promote epithelial-mesenchymal transition (EMT) [22]. Moreover, it has been reported that TAZ promotes migration, invasion, and tumorigenesis of breast cancer cells [23, 24]. The protein levels of TAZ in a panel of breast cancer cell lines correlate with the invasiveness of cancer cells. TAZ protein was also over-expressed in a fraction of breast cancer samples, suggesting that TAZ is an oncoprotein in human cancers [25]. Consistently, TAZ is highly expressed in NSCLC, demonstrating that inactivation of TAZ could be a promising approach for treating NSCLC [26].

In the current study, we determined whether over-expression or depletion of TAZ could govern cell growth, migration, and invasion in NSCLC cells. We also explored whether rottlerin exhibits its anticancer activity via inhibition of TAZ in NSCLC. Our results showed that TAZ was critically involved in the progression of NSCLC. Moreover, we found that rottlerin down-regulated the expression of TAZ, leading to inhibition of tumor growth and motility activity in NSCLC. These findings indicate that rottlerin could be a potential efficient agent for the prevention and treatment of NSCLC.

## RESULTS

### Rottlerin inhibited cell proliferation

To explore whether rottlerin exhibits anti-proliferation function in lung cancer cells, CTG assay was applied for detection of cell growth viability in both SPC-A-1 and A549 lung cancer cells after treatment with different concentrations of rottlerin for 48 h and 72 h. Our results showed that rottlerin significantly inhibited cell growth in time- and dose- dependent manners in both SPC-A-1 and A549 cells (Figure 1B). Specifically, the IC_50_ that caused 50% inhibition of cell growth at 72 h for SPC-A-1 cells was about 2 μM, and around 1 μM for A549 cells. Therefore, in the following studies, we used 2 μM and 1 μM rottlerin treatments for SPC-A-1 and A549 cells, respectively.

### Rottlerin induced apoptosis

It has been known that increased apoptosis could be involved in rottlerin-mediated cell growth inhibition in cancer cells. Thus, we further detected whether rottlerin could trigger apoptosis in lung cells. To this end, we measured the effects of rottlerin treatment on apoptotic cell death using PI-FITC-annexin assay in SPC-A-1 cells treated with 2 μM, 4 μM rottlerin, and in A549 cells with 1 μM, 3 μM rottlerin treatments for 48 hours. We observed that rottlerin treatment stimulated cell apoptosis in dose-dependent manner (Figure 1C). Our finding suggests that rottlerin inhibited cell growth partly due to induction of cell apoptosis by rottlerin in lung cancer cells.

### Rottlerin induced cell cycle arrest

**Figure 2:**
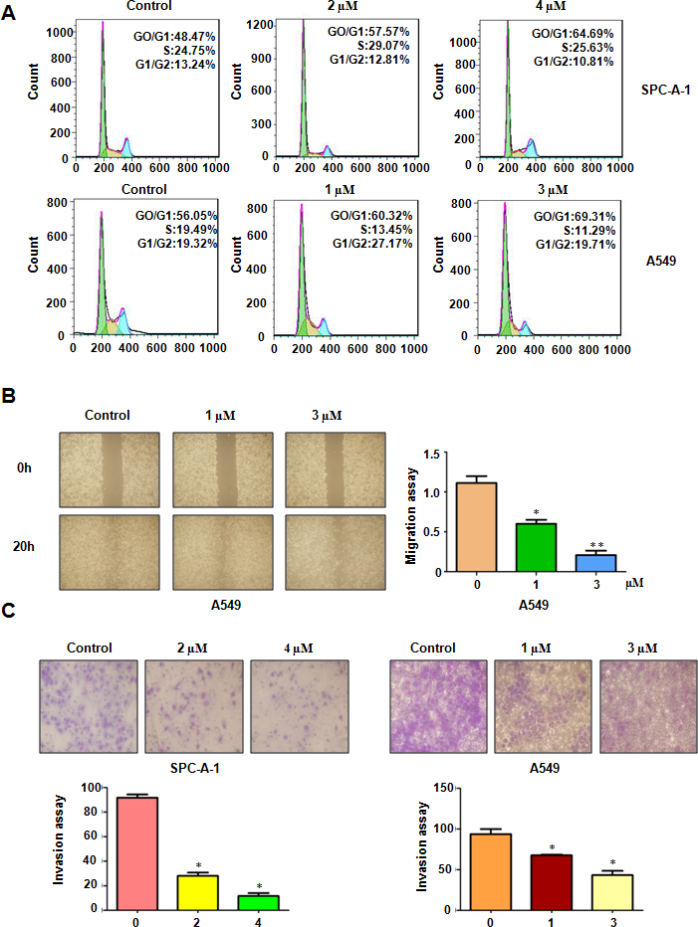
Rottlerin induced cell cycle arrest and inhibited cell migration and invasion on NSCLC (**A**) Cell cycle was analyzed by Fow cytometry. Rottlerin induced NSCLC cell cycle arrest. (**B**) The inhibitory effect of rottlerin on NSCLC migration was detected using wound healing assay in A549 cells. Right panel, Quantitative results are illustrated for left panels. *P < 0.05, vs control group (DMSO treatment). (**C**) Top panel, the inhibitory effect of rottlerin on NSCLC invasion was detected by Transwell chambers assay. Bottom panels, Quantitative results are illustrated for Top panel. *P < 0.05, **< 0.01 vs control.

To ensure whether rottlerin regulates cell cycle in lung cancer cells, we conducted the cell cycle analysis by PI staining and flow cytometry in both SPC-A-1 and A549 cells treated with different concentrations of rottlerin for 48 hours. We found that the cells population in G0/G1 phase was increased from 48.47% to 57.57% to 64.49% in SPC-A-1 cells after 2 μM and 4 μM rottlerin treatments, respectively (Figure 2A). Similarly G0/G1 arrest results were observed in the A549 cells treated with rottlerin (Figure 2A). Altogether, a typical G0/G1 arrest pattern with rottlerin treatment was identified in both SPC-A-1 and A549 cells, demonstrating that rottlerin triggered cell cycle arrest at G0/G1 phase.

### Rottlerin retarded cell migration and invasion

To analyze whether rottlerin could retard the cell motility in lung cancer cells, the wound healing assay using a scratch approach was conducted in A549 cells treated with 1 μM and 3 μM rottlerin for 20 hours. Our wound healing assay discovered that rottlerin significantly decreased cell migration in A549 cells (Figure 2B). To further validate the effect of anti-motility induced by rottlerin in lung cancer cells, invasion assay using matrigel-coated membrane was performed in rottlerin-treated cells. In keeping with the migration result, rottlerin treatment caused decreased penetration of lung cancer cells via the matrigel-coated membrane compared with the control cells (Figure 2C). Taken together, rottlerin has anti-invasive function in lung cancer cells.

### Rottlerin suppressed TAZ expression

**Figure 3:**
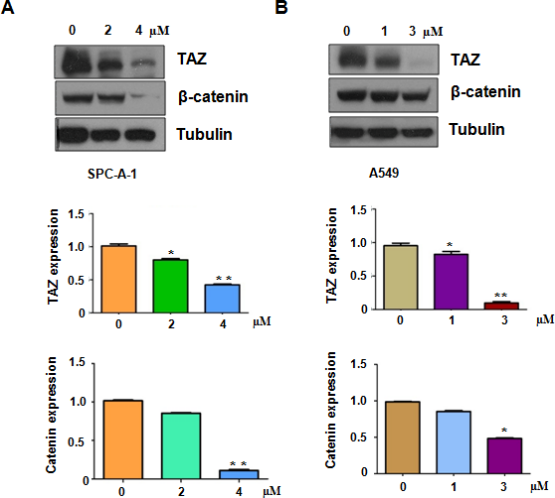
Rottlerin downregulated TAZ expression (**A**) Top panel, the expression of TAZ, β-catenin was determined by western blotting analysis in SPC-A-1 cells. Bottom panels, Quantitative results are illustrated for Top panel. (**B**) Top panel, the expression of TAZ, β-catenin was determined by western blotting analysis in A549 cells. Bottom panels, Quantitative results are illustrated for Top panel.* P < 0.05, **< 0.01 vs control (DMSO treatment).

Emerging evidence has confirmed that TAZ oncoprotein plays a pivotal role in lung tumorigenesis. To determine whether TAZ was involved in rottlerin-mediated anti-tumor activities, we measured the expression of TAZ in SPC-A-1 and A549 cells after rottlerin treatments. Our Western blotting analysis data showed that rottlerin decreased the TAZ protein level in both lung cancer cells (Figure 3A and 3B). We also detected the expression of β-catenin, which is one of TAZ downstream target genes [27–29]. We found that rottlerin down-regulated the expression of β-catenin in both SPC-A-1 and A549 cells (Figure 3A and 3B). Our findings revealed that rottlerin suppressed TAZ expression in lung cancer cells.

### Over-expression of TAZ rescued rottlerin-induced cell growth inhibition

**Figure 4:**
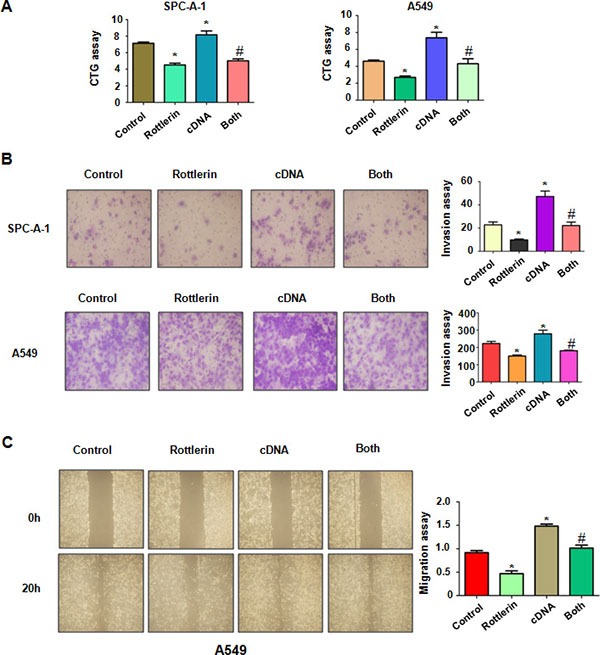
The effect of TAZ overexpression on cell growth and invasion (**A**) CTG assay was used to detect the effect of TAZ overexpression in combination with rottlerin treatment on NSCLC proliferation. *P < 0.05, **< 0.01 vs control. #p < 0.05 compared with rottlerin treatment or TAZ cDNA transfection. Control: pcDNA3.1 transfection; cDNA : TAZ cDNA; Both : TAZ cDNA + rottlerin. (**B**) Left panel, invasion assay was performed in NSCLC after TAZ cDNA transfection and rottlerin treatment. Right panel, quantitative results are illustrated for left panel. (**C**) Left panel, the wound healing assay was conducted to detect the cell migration in NSCLC after TAZ cDNA transfection and rottlerin treatment. Right panel, quantitative results are illustrated for left panel.

To dissect whether rottlerin exerts its anti-tumor activity partly through inactivation of TAZ in lung cancer cells, SPC-A-1 and A549 cells were transfected with TAZ cDNA or empty vector as control. We found that overexpression of TAZ promoted cell growth in both SPC-A-1 and A549 cells (Figure 4A). Importantly, the cell growth inhibition triggered by rottlerin treatment was rescued by over-expression of TAZ in both lung cancer cell lines (Figure 4A). These results indicated that rottlerin inhibited cell growth in part due to downregulation of TAZ in lung cancer cells.

### Over-expression of TAZ promoted cell motility

**Figure 5:**
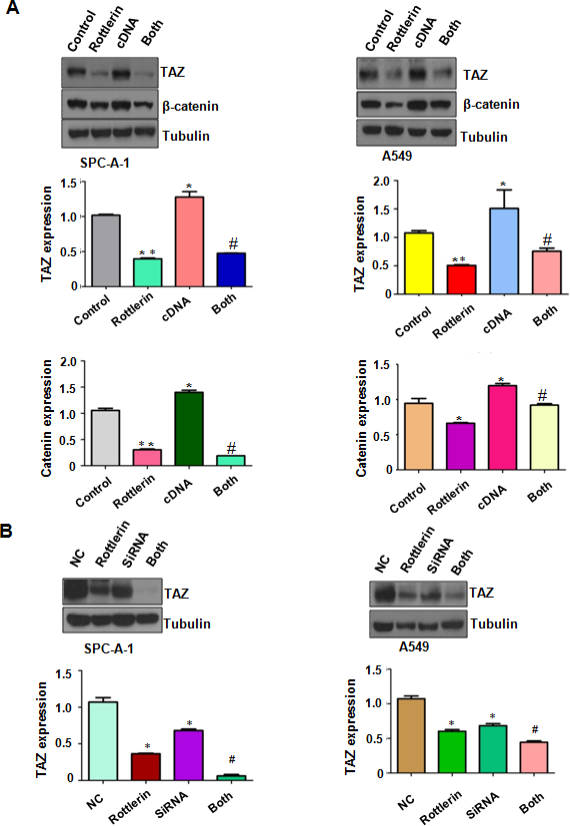
Down-regulation of TAZ by its siRNA promoted rottlerin-induced TAZ inhibition (**A**) Top panel: The expression of TAZ and β-catenin was detected by Western blotting in NSCLC with TAZ cDNA transfection and rottlerin treatment. Bottom panels, Quantitative results are illustrated for Top panel.*P < 0.05, **< 0.01 vs control. #p < 0.05 compared with rottlerin treatment or TAZ cDNA transfection. Control: pcDNA3.1 transfection; cDNA : TAZ cDNA; Both : TAZ cDNA + rottlerin. (**B**) Top panel: The expression of TAZ was detected by Western blotting in NSCLC with TAZ siRNA transfection and rottlerin treatment. Bottom panels, Quantitative results are illustrated for Top panel.*P < 0.05, **< 0.01 vs control. #p < 0.05 compared with rottlerin treatment or TAZ siRNA transfection. Control: control siRNA; siRNA : TAZ siRNA; both : TAZ siRNA + rottlerin.

Next, we detected whether TAZ could enhance cell motility in lung cancer cells. We found that overexpression of TAZ enhanced cell invasion in both lung cancer cells (Figure 4B). Consistently, the wound healing assay showed that over-expression of TAZ caused increased numbers of cells migrating across the wound (Figure 4C). Furthermore, our Western blotting assay demonstrated that overexpression of TAZ by its cDNA transfection rescued the rottlerin-mediated inhibition of TAZ in lung cancer cells (Figure 5A). Our results also showed that the expression of β-catenin is also rescued by over-expressing TAZ compared with rottlerin treatment alone. These results provide the evidence that rottlerin could exert anti-tumor activity through down-regulation of TAZ and its target gene β-catenin.

### Down-regulation of TAZ by its siRNA promoted rottlerin-induced anti-tumor activity

**Figure 6:**
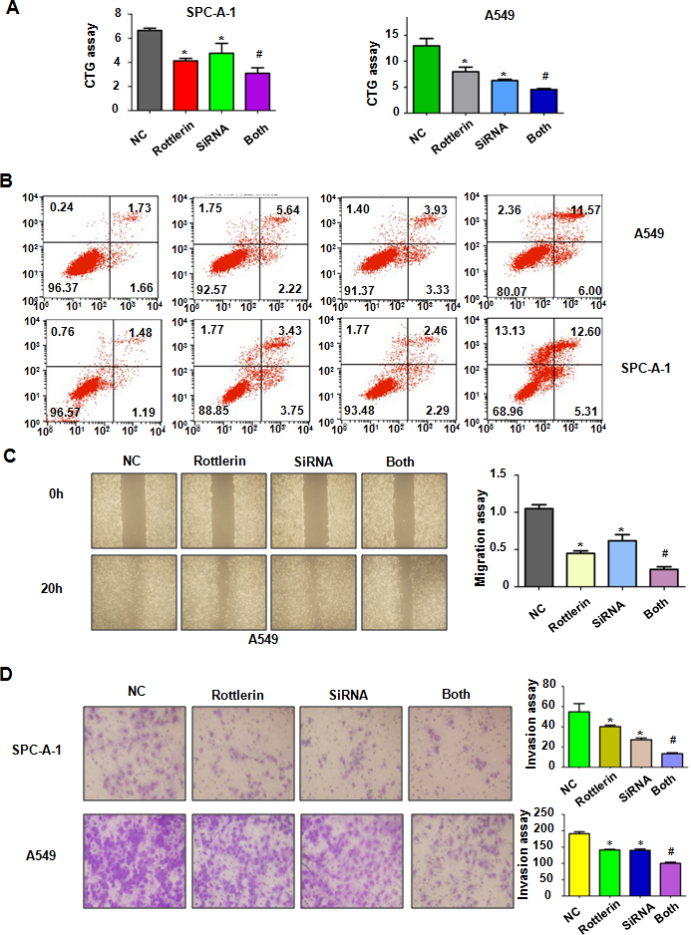
The effect of TAZ downregulation on cell growth, apoptosis, migration, and invasion (**A**) CTG assay was used to detect the effect of TAZ siRNA in combination with rottlerin treatment on NSCLC proliferation. Both: rottlerin + TAZ siRNA. *P < 0.05, **< 0.01 vs control. #p < 0.05 compared with rottlerin treatment or TAZ siRNA transfection. Control: control siRNA; siRNA : TAZ siRNA; both : TAZ siRNA + rottlerin. (**B**) Cell apoptosis treated with TAZ siRNA in combination with rottlerin treatment on NSCLC was determined by Flow cytometry. (**C**) Left panel, the wound healing assay was conducted to detect the cell migration in NSCLC after TAZ siRNA transfection and rottlerin treatment. Right panel, quantitative results are illustrated for left panel. (**D**) Left panel, invasion assay was performed in NSCLC after TAZ siRNA transfection and rottlerin treatment. Right panel, quantitative results are illustrated for left panel.

To deeper investigate the role of TAZ in rottlerin-mediated tumor suppressive activity, we down-regulated the expression of TAZ by its siRNA transfection in lung cancer cells treated with rottlerin. We found that TAZ siRNA transfection significantly downregulated TAZ expression in both lung cancer cells (Figure 5B). Moreover, TAZ siRNA transfection enhanced inhibition of TAZ expression induced by rottlerin (Figure 5B). Our CTG assay showed that depletion of TAZ suppressed cell growth in lung cancer cells (Figure 6A). Notably, cell growth was significantly inhibited by rottlerin combined with TAZ siRNA transfection (Figure 6A). Furthermore, TAZ siRNA-transfected cells were significantly more sensitive to spontaneous and rottlerin-induced apoptosis (Figure 6B). Strikingly, we identified that down-expression of TAZ suppressed migration and invasion in A549 cells (Figure 6C and 6D). Depletion of TAZ in combination with rottlerin treatment retarded cell migration and invasion to a greater degree compared with rottlerin treatment alone (Figure 6C and 6D). In summary, depletion of TAZ promoted rottlerin-induced anti-tumor activity in lung cancer cells.

## DISCUSSION

There is growing evidence that TAZ plays an oncogenic role in a wide range of human cancers including NSCLC [30–34]. TAZ was observed to have highly expression in NSCLC cells [26]. Overexpression of TAZ promoted cell proliferation and transformation, while knockdown of TAZ expression inhibited cell proliferation *in vitro* and tumor growth in mice [26]. In line with this finding, knockdown of TAZ retarded *in vitro* cellular migration and transplantation of metastasis in lung cancer cells [35]. Clinicopathologically, overexpression of TAZ was associated with lung adenocarcinoma, poorer differentiation, lymph node metastasis, and poorer prognosis, suggesting that TAZ expression is a prognostic indicator for worse survival in patients with resected NSCLC [36]. Moreover, Hsu et al. found that angiomotin decreased lung cancer progression through sequestering YAP/TAZ expression [37]. Accordingly, overexpression of TAZ rendered lung cancer cells with EGFR (epidermal growth factor receptor) mutation resistant to gefitinib, indicating that combinational targeting on both EFGR and TAZ could enhance the efficacy of EGFR tyrosine kinase inhibitors to overcome the acquired resistance of NSCLC [38]. In concert with these observations, our results demonstrated that up-regulation of TAZ promoted cell growth, migration, and invasion, whereas down-regulation of TAZ suppressed cell growth and motility activity in lung cancer cells. Taken together, our study provides the rationale for the development of specific TAZ inhibitors as potential anti-cancer agents.

A wealth of evidence has emerged that deactivation of the Hippo pathway and up-regulation of TAZ were observed in a wide spectrum of human cancers, suggesting that inactivation of TAZ could be a promising strategy for the treatment of cancers [39]. For example, microRNA-129-5p inhibited ovarian cancer cell proliferation and survival through suppression of TAZ [40]. Several small molecules including dasatinib, statins and pazopanib have been reported to inhibit the nuclear localization and gene expression of YAP and TAZ [41]. Moreover, pazopanib triggered proteasomal degradation of YAP/TAZ in breast cancer cells [41]. Moreover, statins inhibited HMG-CoA (3-hydroxy-3-methyl glutaryl coenzyme A) reductase and led to impaired geranylgeranylation of RHOA, resulting in the inactivation of YAP/TAZ [42, 43]. Due to that higher concentrations of these drugs in blood could be a risk factor for adverse effect, development of new agents from natural compounds might be a better approach for treating human cancers. In the current study, we found that rottlerin inhibited TAZ expression, leading to anti-tumor activity in NSCLC. Our study thus offers a new strategy to treat NSCLC via rottlerin as a single agent or in combination with chemotherapeutic agents.

In summary, our study reveals that rottlerin exerted its tumor suppressive function via inactivation of TAZ in NSCLC cells, suggesting that inhibition of TAZ by rottlerin could be a promising strategy for the prevention and therapy of NSCLC. To gain further insight into the role of TAZ in rottlerin-mediated anti-tumor activity, more investigations are required to explore how rottlerin governs the expression of TAZ. *In vivo* experiment is necessary to determine whether rottlerin suppresses tumor growth via targeting TAZ in mice. Moreover, it is pivotal to investigate whether rottlerin could sensitize the efficacy of chemotherapeutic drugs to achieve the better treatment outcome in NSCLC. In summary, down-regulation of TAZ by rottlerin could be a potential effective approach to treat NSCLC.

## MATERIALS AND METHODS

### Cell culture and reagents

Human lung cancer cell lines SPC-A-1 and A549 were cultured in DMEM supplemented with 10% fetal bovine serum and 1% penicillin and streptomycin. Primary antibodies against TAZ and P-YAP (127) were obtained from Santa Cruz Biotllechnology (Santa Cruz, CA). All secondary antibodies were obtained from Thermo Scientific. Lipofectamine 2000 was purchased from Invitrogen. Monoclonal anti-β-actin, monoclonal anti-tubulin, rottlerin (CAS number R5648, ≥ 85% rottlerin) and CTG were obtained from Sigma-Aldrich (St.Louis, MO). Rottlerin was dissolved in DMSO to make a 30 mM stock solution and was added directly to the medium at different concentrations. In the control group, cells were treated with 0.1% DMSO.

### Cell viability assay

Cells were seeded in 96-well plate for overnight. Then, cells were treated with different concentrations of rottlerin. After two days and three days, 20 μl of the CTG (5 mg/ml) solution was added to each well and incubated for 10 min at 37°C. Subsequently, the reaction mixture was detected by the microplateau at 490 nm.

### Cell apoptosis analysis

Cells were seeded in six-well plate for overnight and treated with various concentrations of rottlerin for 48 h. Then, cells were collected and washed with PBS. Subsequently, cells were resuspended in 500 μl binding buffer including Propidium iodide (PI) and FITC-conjugatedanti-Annexin V antibody. Apoptosis was measured by a FACScalibur flow cytometer (BD, USA) as described before [44].

### Cell cycle analysis

Cells were seeded in a 6-well plate for overnight. Then, cells were treated with different concentrations of rottlerin for 48 h. Subsequently, cells were harvested and washed with cold PBS. Suspended cells with 70% cold alcohol were kept at 4°C overnight. Then, cells were washed with cold PBS, and re-suspended at 1 × 10^6^ cells/ml in PBS. Cells were further incubated with 0.1 mg/ml RNase I and 50 mg/ml Propidium iodide (PI) at 37°C for 30 min. Cell cycle was measured by a FACScalibur flow cytometer (BD, USA).

### Cell scratch assays

A549 cells were cultured in 6-well plate. After cells converged almost 100%, the supernatant was absorbed and the cells were scratched by a yellow pipette tips. The cells were washed with PBS and added medium with rottlerin. The scratched area was photographed with microscope at 0 h and 20 h, respectively [45].

### Cell invasion assay

To determine the cell invasion, Transwell assay was performed in SPC-A-1 and A549 cells treated with rottlerin or TAZ transfection or combination [45]. Transfected cells were cultured in the upper chamber with 200 μl serum-free medium. The complete medium was added in the under chamber with the same concentration of rottlerin. After 24 h, the membrane of the chamber was strained with Giemsa and photographed with a microscope.

### Transfection

Cells were seeded into 6-well plate and transfected with TAZ cDNA or TAZ siRNA or empty vector using lipofectamine 2000 following the instruction's protocol [46]. TAZ siRNA: sense 5′-GCA UCU UCG ACA GUC UUC UTT-3′; antisense 5′-AGA AGA CUG UCG AAG AUG CTT-3′. After the transfection, the cells were subjected to further analysis as described under the results sections.

### Western blotting analysis

The cells were washed by PBS and lysed with protein lysis buffer (50 mmol/l Tris (pH 7.5), 100 mmol/l NaCl, 1 mmol/l EDTA, 0.5% NP40, 0.5% Triton X-100, 2.5 mmol/l sodium orthovanadate, 10 ll/mL protease inhibitor cocktail and 1 mmol/l PMSF). BCA Protein Assay kit (Thermo Scientific, MA) was used to measure the concentrations of the proteins. Protein samples were separated by electrophoresis in gel and then transferred onto a membrane, and further incubated with primary antibody at 4°C overnight. After membranes were washed with TBST for three times and incubated with second antibody at room temperature for 1 h. Then the expression of protein was measured by electrochemiluminescence assay.

### Statistical analysis

All statistical analyses were analyzed by GraphPad Prism 5.0 (Graph Pad Software, La Jolla, CA). ANOVA was used to evaluate statistical significance. The data were presented as means ± SD. *P* < 0.05 was considered as significant.
